# Smoking and High‐Altitude Exposure Affect Intrinsic Neural Activity: A fMRI Study of Interactive Effects

**DOI:** 10.1111/adb.70042

**Published:** 2025-04-24

**Authors:** Qingqing Lv, Minghe Wang, Chunxiao Bu, Junjie Liao, Kefan Wang, Hui Xu, Xijuan Liang, Ning Zheng, Liangjie Lin, Longyao Ma, Weijian Wang, Zhen Ma, Meiying Cheng, Xin Zhao, Lin Lu, Yong Zhang

**Affiliations:** ^1^ Department of Radiology Third Affiliated Hospital of Zhengzhou University Zhengzhou China; ^2^ ZhengZhou Health Vocational College Zhengzhou China; ^3^ Department of Magnetic Resonance Imaging First Affiliated Hospital of Zhengzhou University Zhengzhou China; ^4^ Department of Radiology Qinghai Provincial People's Hospital Xining China; ^5^ Clinical and Technical Support Philips Healthcare Beijing China

**Keywords:** amplitude of low‐frequency fluctuation, high‐altitude exposure, interactive effect, nicotine addiction, resting‐state functional magnetic resonance imaging, smoking

## Abstract

Smoking and high‐altitude (HA) exposure both adversely affect human health, with smoking linked to various cancers and high‐altitude environments causing physiological and neurological changes. Although the effects of smoking and HA exposure on brain structure and function have been studied separately, their combined impact is still rarely explored. This study aims to investigate the interactive effects of smoking and HA exposure on intrinsic brain activity using the resting‐state functional magnetic resonance imaging (rs‐fMRI) analysed by the amplitude of low‐frequency fluctuations (ALFF) method. We used a mixed sample design, including four groups: (i) HA smokers (*n* = 22); (ii) HA nonsmokers (*n* = 22); (iii) sea‐level (SL) smokers (*n* = 26); and (iv) SL nonsmokers (*n* = 26), for a total of 96 male participants. All subjects underwent resting‐state functional magnetic resonance imaging. ALFF was used to assess differences in brain activity among the four groups. Two‐way analysis of variance (ANOVA) was conducted to analyse the effects of smoking, high‐altitude exposure and their interaction on ALFF. As for the main effect of smoking, elevated ALFF was found in the right superior frontal gyrus, right middle frontal gyrus, right inferior frontal gyrus, right middle cingulate cortex and right precentral gyrus. As for the main effect of HA exposure, elevated ALFF was found in the right putamen, right insula, right inferior frontal gyrus, right middle temporal gyrus, right precentral gyrus, right inferior temporal gyrus and right fusiform. A significant interaction effect between smoking and HA exposure was observed in the right precentral gyrus. Post hoc analysis for the right precentral gyrus showed significantly increased ALFF in groups including HA versus SL smokers; HA versus SL nonsmokers; and HA smokers versus HA nonsmokers. Our findings demonstrate that both smoking and HA exposure independently influence spontaneous brain activity, with a significant interaction between the two factors in modulating brain function. These results offer a neuroimaging‐based perspective on substance addiction in high‐altitude populations and contribute to a deeper understanding of high‐altitude adaptation.

## Graphical abstract

This study used ALFF analysis of rs‐fMRI to explore the effects of smoking and high‐altitude (HA) exposure on brain activity. Both factors independently increased ALFF in several brain regions, with a significant interaction in the right precentral gyrus. These findings offer neuroimaging evidence for addiction mechanisms and HA adaptation.

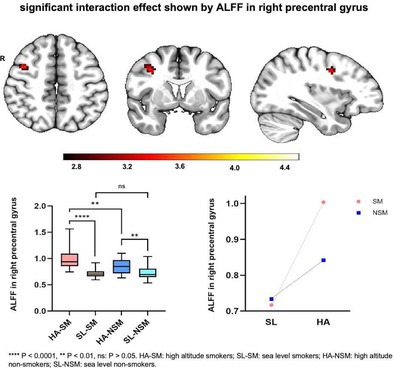

## 1 Introduction

Smoking and exposure to high altitudes can both have numerous adverse effects on the human body, leading to various health problems. Research indicates that smoking is associated with increased risk of various cancers, including lung, oesophageal and bladder cancers [1]. The World Health Organization reports that about 6 million people die from smoking‐related diseases every year, of which > 5 million people die from direct use of tobacco products [2]. Nicotine, a main component of tobacco, is the primary reason for tobacco addiction. Tobacco addiction is a chronic mental disorder characterized by impaired inhibitory control and compulsive tobacco seeking and smoking [3]. Despite smokers' awareness of the severe long‐term consequences of smoking, the failure rate for smoking cessation is as high as 85% [4]. High‐altitude (HA) residents constitute a distinct population, characterized by inhabiting regions exceeding 1000 m above sea level. High altitudes can lead to reduced oxygen concentration, resulting in hypoxic environments. Prolonged exposure to low oxygen levels has significant effects on health, leading to physiological adaptations known as high‐altitude adaptation (HAA), a process in which multiple physiological systems synergize [5]. For instance, in order to ensure efficient blood gas exchange, the low oxygen ventilatory response increases oxygen intake, whereas pulmonary vasoconstriction enhances blood perfusion [6]. Additionally, HAA may exert feedback effects on corresponding control centres within the brain. Reportedly, the hypoxic environment induced by high altitudes can impact the structure and metabolism of the brain, resulting in decreased neuronal processing capabilities and subsequent impairments in cognition, memory and learning among high‐altitude residents [7].

In recent years, numerous studies using structure and functional magnetic resonance imaging (fMRI) have shown that sustained exposure to smoking and HA impacts the structure and intrinsic activity of the brain. Structurally, Yang et al. [8] found that long‐term smokers have grey matter (GM) volume decrease in the bilateral prefrontal cortex and left insular and GM volume increase in the right lingual cortex and left occipital cortex. A meta‐analysis found that substance addiction subjects including tobacco showed decreased grey matter volume in the orbitofrontal cortex, insula, anterior cingulate and striatum [9]. As for HA, it has been reported that residents of high‐altitude regions exhibit reduced grey matter volume in the bilateral prefrontal cortex and bilateral insular cortex, possibly related to anaerobic metabolic byproducts of brain tissue during chronic hypoxia [10]. Chen et al. [11] found that chronic hypoxic exposure at high altitudes leads to a decrease in grey matter volume in the left caudate nucleus. Functionally, amplitude of low‐frequency fluctuations (ALFF) serves as an index reflecting the strength of spontaneous neuronal activity and elucidating neural processes during resting states [12]. Previous study demonstrated that increased ALFF in superior frontal gyrus and middle frontal gyrus as well as decreased ALFF in calcarine sulcus were observed in smokers compared with nonsmokers in sea level (SL) regions [13]. Also, a HA‐related functional MRI study discovered that the HA group had lower ALFF values in the cerebellum, putamen, orbital inferior frontal gyrus and precuneus but higher ALFF values of the fusiform gyrus, inferior temporal gyrus and superior frontal gyrus, compared to the low‐altitude group [14].

Given that both smoking and hypoxia can independently affect cerebral oxygenation, vascular function and neurotransmitter balance, it is crucial to explore their combined impact on brain activity. Understanding these interactions may provide novel insights into addiction mechanisms and cognitive‐motor function alterations in individuals exposed to both factors. Despite the abundance of neuroimaging studies, there has yet to be any research exploring interactive effects in intrinsic brain activity between smoking and HA. To cover these gaps, the subjects were divided into four groups (HA smokers/nonsmokers and SL smokers/nonsmokers), and the ALFF method was used to analyse the independent and interactive effects of smoking and HA exposure on intrinsic brain activity.

## 2 Materials and Methods

### 2.1 Participants

Forty‐four HA and 52 SL residents were recruited and divided into four groups, including (i) HA smokers (HA‐SM, *n* = 22); (ii) HA nonsmokers (HA‐NSM, *n* = 22); (iii) SL smokers (SL‐SM, *n* = 26); and (iv) SL nonsmokers (SL‐NSM, *n* = 26) in this study.

The inclusion criteria for participants were as follows: All participants were male and right‐handed, all HA participants were local residents from Qinghai Province of China, residing in high‐altitude for over 20 years and all SL participants were sea‐level residents who had not spent more than 7 days in high‐altitude areas. Smoker participants were required to meet the following criteria: (1) smoking at least 1 cigarette daily for more than 2 years and (2) meeting the nicotine dependence criteria according to the DSM‐IV [15]. The severity of nicotine dependence was assessed using the Fagerström Test for Nicotine Dependence (FTND) score [16] and pack‐year (smoking years × number of cigarettes smoked per day/20). Nonsmoker participants included individuals who were currently nonsmokers and had smoked fewer than five cigarettes in their lifetime.

Exclusion criteria for participants were (1) any neurological or psychiatric disorders or other medical conditions; (2) evidence of drug abuse (excluding nicotine) or substance abuse according to urine tests and self‐reporting; and (3) any contraindications to magnetic resonance imaging (MRI).

This study was approved by the Ethics Committee of Qinghai Provincial People's Hospital, and written informed consent was obtained from all participants.

### 2.2 Imaging Acquisition Measures

All participants underwent fMRI using a Siemens MAGNETOM Skyra 3.0T scanner equipped with a dedicated 16‐channel head coil. During the scanning procedure, participants were instructed to keep their eyes closed (but remain awake) and relax their mind. We utilized soft padding and earplugs to minimize head motion and machine noise. Participant wakefulness was monitored through verbal communication during the scan. Blood oxygen level‐dependent (BOLD) resting‐state fMRI (rs‐fMRI) utilizes an echo‐planar imaging sequence with the following parameters: repetition time = 2000 ms, echo time = 30 ms, flip angle = 80°, field of view = 240 × 240 mm^2^, matrix = 64 × 64, slice thickness = 4 mm and 180 dynamic volumes were obtained. The scan was set to axial orientation and positioned along the anterior commissure–posterior commissure (AC‐PC) line, and the total scan time was 360 s.

### 2.3 Data Analysis

The rs‐fMRI data were processed using the DPARSF tool within DPABI (http://rfmri.org/DPABI), based on Matlab R2018a. The processing steps were as follows: conversion of data from DICOM format to 4D NIfTI format; (2) removal of the initial five volumes; (3) slice timing correction and realignment; (4) normalizing to the EPI template in standard Montreal Neurological Institute (MNI) space using linear and nonlinear transformations and resampling with a resolution of 3 × 3 × 3 mm^3^; (5) several spurious variances, including signals from white matter, cerebrospinal fluid, and 24 head motion parameters were regressed out using multiple linear regression analysis [17]; (6) calculation of Frame‐wise Displacement (FD) for each time point [18] and exclusion of data with a mean FD > 0.5 mm [19]; (7) spatial smoothing using a 6‐mm full‐width at half‐maximum Gaussian kernel, followed by detrending; and (8) bandpass filtering with a frequency range of 0.01–0.08 Hz.

ALFF was calculated using REST (rs‐fMRI Data Analysis Toolkit). The time series of each voxel was transformed into frequency domain using fast Fourier transform, resulting in the power spectrum. The square root of the power spectrum was then calculated within the specified frequency range (0.01–0.08 Hz) to obtain mean amplitude, known as ALFF, at each voxel. To standardize the data, the ALFF value of each voxel was divided by the global mean ALFF value.

### 2.4 Statistical Analysis

The two‐sample *t* test or the Mann–Whitney *U* test were used to compare the demographic information (age and education level) and clinical characteristics (smoking years, pack year and FTND score) between SM and NSM subjects, as well as between HA and SL subjects, with differences considered significant at *p* < 0.05. We used the Anatomical Automatic Labeling (AAL) template to identify brain regions. A two‐way analysis of variance (ANOVA) was performed using the full factorial model in SPM12 to analyse the effects of HA exposure and smoking on whole‐brain ALFF maps, with education, age and mean FD included as covariates. Gaussian random field theory (GRF) was used for multiple comparison correction with voxel level *p* < 0.005 and cluster level *p* < 0.05. The HA main effect map was shown with threshold at voxel level *p*_*FWE*_ (family wise error corrected *p* value) < 0.001 and cluster level *p* < 0.05, and the minimum cluster size was set to 100 for better visualization. The primary reason for using different voxel‐level *p* values was to ensure clearer visualization of the results. When analysing the main effect of smoking, we initially applied a stricter threshold (*p* < 0.001), but the results were too sparse to be meaningfully visualized. Therefore, we opted for a slightly more lenient threshold (*p* < 0.005) to better illustrate the spatial distribution of smoking‐related brain activity changes while still controlling for multiple comparisons at the cluster level. We note that the threshold of *p* < 0.005 is not as conservative as *p* < 0.001, and therefore, we do not claim strong Type I error control for these exploratory analyses. Each cluster showing a significant interaction effect of HA and smoking on ALFF was designated as a region of interest (ROI). ALFF values were then extracted from the ROI, and post hoc comparisons were carried out using two‐sample *t* tests to analyse interaction effects, with significance set at *p* < 0.0125 (Bonferroni corrected).

### 2.5 Correlation Analyses

To examine the association of ALFF change with nicotine addiction severity, we carried out Pearson correlation analyses between ALFF values of brain regions (altered in interaction effect analyses) and clinical data (age onset of smoking, smoking years, pack‐year and FTND score), while controlling for the effect of age and education.

## 3 Results

### 3.1 Demographics and Clinical Characteristics

Demographic and clinical characteristics were shown in Table 1. There were no significant differences in age and education years between smokers and nonsmokers or between HA and SL groups (details in Table 1). HA and SL smokers did not have difference in age onset of smoking (*z* = −1.519, *p* = 0.129), smoking years (*t* = 1.041, *p* = 0.306), pack‐year (*z* = −0.394, *p* = 0.694) and FTND (*t* = 1.011, *p* = 0.317).

**TABLE 1:** Demographics and clinical characteristics.

Demographics and clinical characteristics	HA (*n* = 44)		SL (*n* = 52)		Comparison	
	SM	NSM	SM	NSM	SM vs. NSM	HA vs. SL
Sex (M/F)	22/0	22/0	26/0	26/0	—	—
Age (years)	39.14 ± 7.17	38.95 ± 9.78	40.35 ± 6.58	39.12 ± 5.58	*t* = 0.505 *p* = 0.614	*t* = −0.448 *p* = 0.656
Education (years)	14.41 ± 1.97	14.91 ± 2.04	14.15 ± 2.41	14.31 ± 2.40	*z* = −1.405 *p* = 0.160	*z* = −1.002 *p* = 0.316
Age onset of smoking	19.64 ± 5.21	—	20.42 ± 3.24	—	—	*z* = −1.519 *p* = 0.129
Smoking years	28.27 ± 9.46	—	25.92 ± 5.18	—	—	*t* = 1.041 *p* = 0.306
Pack‐year	26.61 ± 13.45	—	29.18 ± 12.96	—	—	*z* = −0.394 *p* = 0.694
FTND	5.14 ± 2.01	—	4.42 ± 2.74	—	—	*t* = 1.011 *p* = 0.317

*Note:* Data represent mean ± SD.

Abbreviations: F, female; FTND, Fagerström Test for Nicotine Dependence; HA, high altitude; M, male; NSM, nonsmokers; peak‐year, years of smoking × cigarettes smoked per day/20; SL, sea level; SM, smokers.

### 3.2 Main Effects of Smoking and HA Exposure on ALFF

**FIGURE 1:**
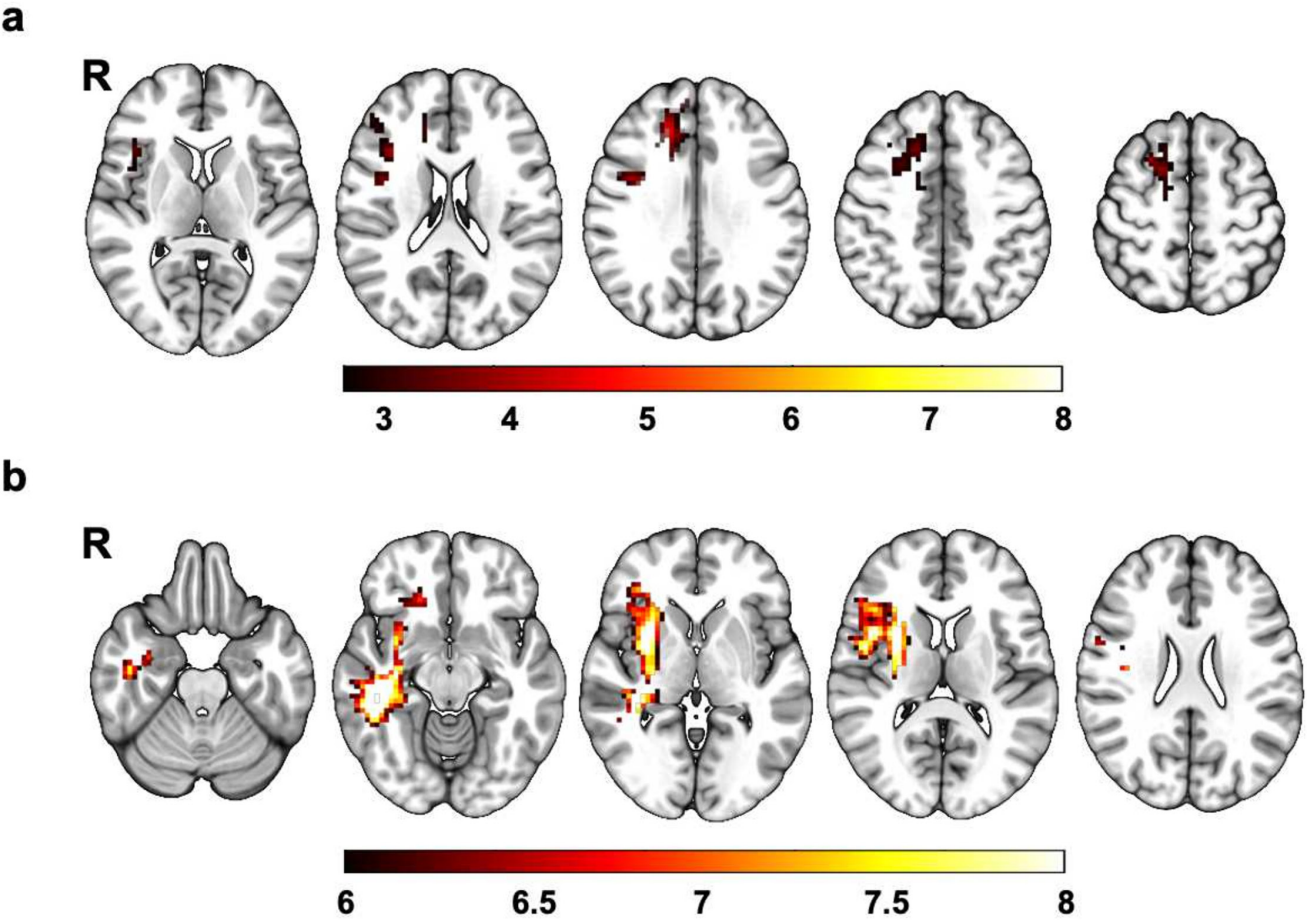
The main effects of HA and smoking. (a) The main effects of smoking, GRF corrected, *p* voxel < 0.005 and *p* cluster < 0.05; (b) the main effects of HA, GRF corrected, *p*_*FWE*_ voxel < 0.001, *p* cluster < 0.05 and cluster size > 100. ‘R’ means the side is to the right.

As for the main effect of smoking, elevated ALFF was found in the right superior frontal gyrus, right middle frontal gyrus, right inferior frontal gyrus, right middle cingulate cortex and right precentral gyrus (Figure 1a and Table 2). As for the main effects of HA, HA exposure group exhibited elevated brain spontaneous fluctuations than SL group in the right putamen, right insula, right inferior frontal gyrus (triangular and opercular part), right middle temporal gyrus, right precentral gyrus, right inferior temporal gyrus and right fusiform (Figure 1b and Table 2).

**TABLE 2:** Significant group differences in amplitude of low‐frequency fluctuation.

Brain region	MNI coordinate			Cluster size	Peak *T* value
	*X*	*Y*	*Z*		
Interaction effect					
Precentral gyrus_R	36	0	40	34	3.31
Main effect of smoking (SM > NSM)					
Superior frontal gyrus_R	17	29	36	226	4.85
Middle frontal gyrus_R	21	31	40	87	3.82
Inferior frontal gyrus (triangular part)_R	40	22	14	44	4.08
Middle cingulate cortex_R	14	26	35	36	4.75
Inferior frontal gyrus (opercular part)_R	37	20	15	36	3.70
Precentral gyrus_R	39	3	34	31	4.19
Main effect of HA (HA > SL)					
Putamen_R	30	9	6	218	9.63
Insula_R	33	6	9	134	7.80
Inferior frontal gyrus (triangular part)_R	42	24	9	88	7.68
Middle temporal gyrus_R	54	−39	−12	84	7.00
Inferior frontal gyrus (opercular part)_R	45	9	9	76	7.81
Precentral gyrus_R/Rolandic operculum	42	−6	18	75	7.82
Inferior temporal gyrus_R	42	15	21	73	7.03
Fusiform_R	45	−24	−18	39	8.22

Abbreviations: MNI, Montreal Neurological Institute; R, right.

### 3.3 Interaction Effects

**FIGURE 2:**
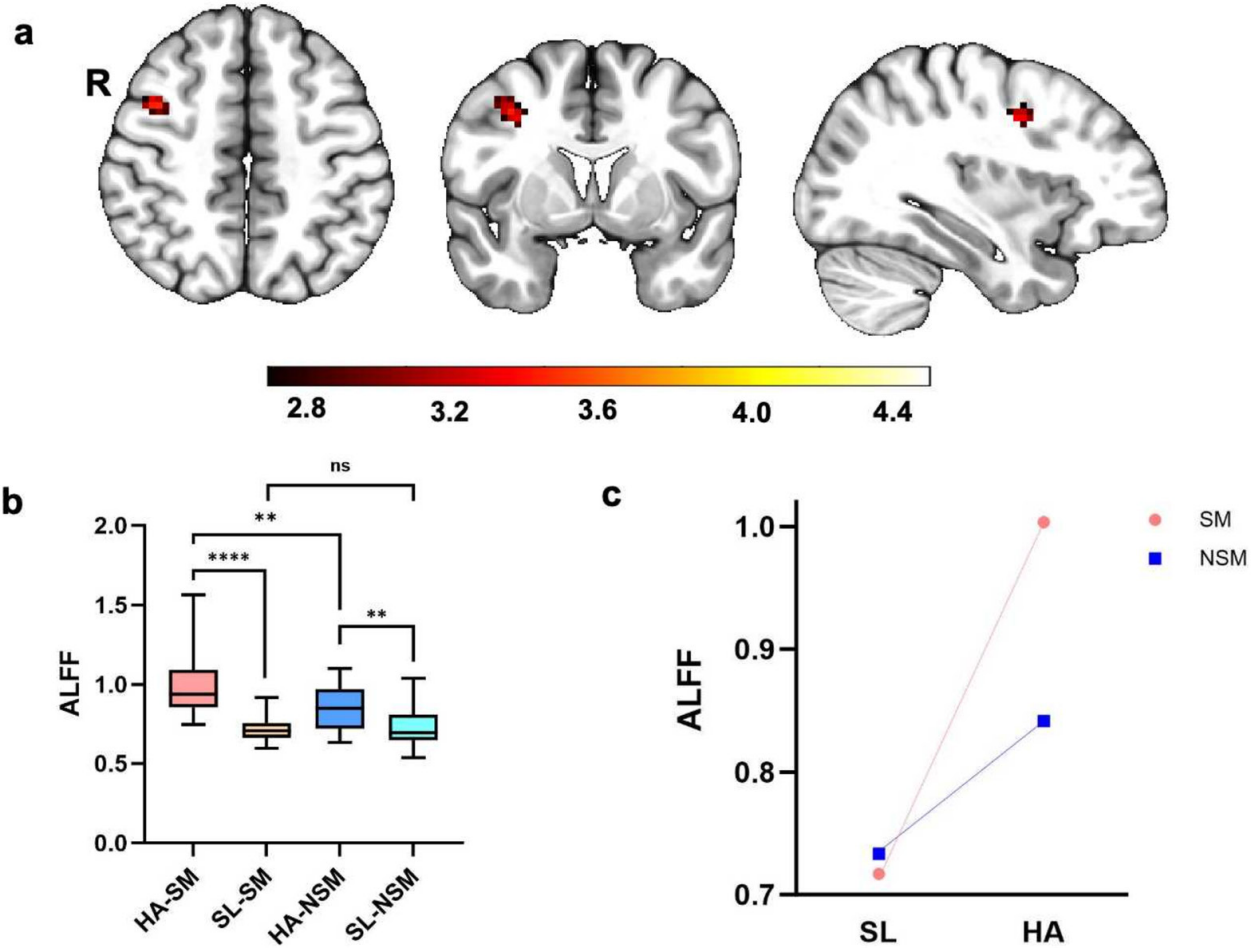
The interaction effect of smoking and HA. (a) A significant interaction effect shown by ALFF in right precentral gyrus using two‐way ANOVA (GRF corrected, *p* voxel < 0.005 and *p* cluster < 0.05). (b, c) Planned post hoc analysis of the right precentral gyrus among the four groups. The vertical bar indicates the maximum and minimum across subjects. ****p < 0.0001, **p < 0.01, ^ns^ *p* > 0.05. HA‐SM, high‐altitude smokers; HA‐NSM, high‐altitude nonsmokers; SL‐NSM, sea‐level nonsmokers; SL‐SM, sea‐level smokers.

The interactive effect of smoking and HA was found in the right precentral gyrus (Figure 2a). Post hoc analysis for the right precentral gyrus showed significantly increased ALFF in HA smokers compared to SL smokers (*p* < 0.0001) and significantly increased ALFF in HA nonsmokers compared to SL nonsmokers (*p* = 0.0096) and significantly increased ALFF in HA smokers compared to HA nonsmokers (*p* = 0.007). No significant difference was found between SL smokers and SL nonsmokers (*p* = 0.5923) (Figure 2b,c).

### 3.4 Correlation Analysis

**FIGURE 3:**
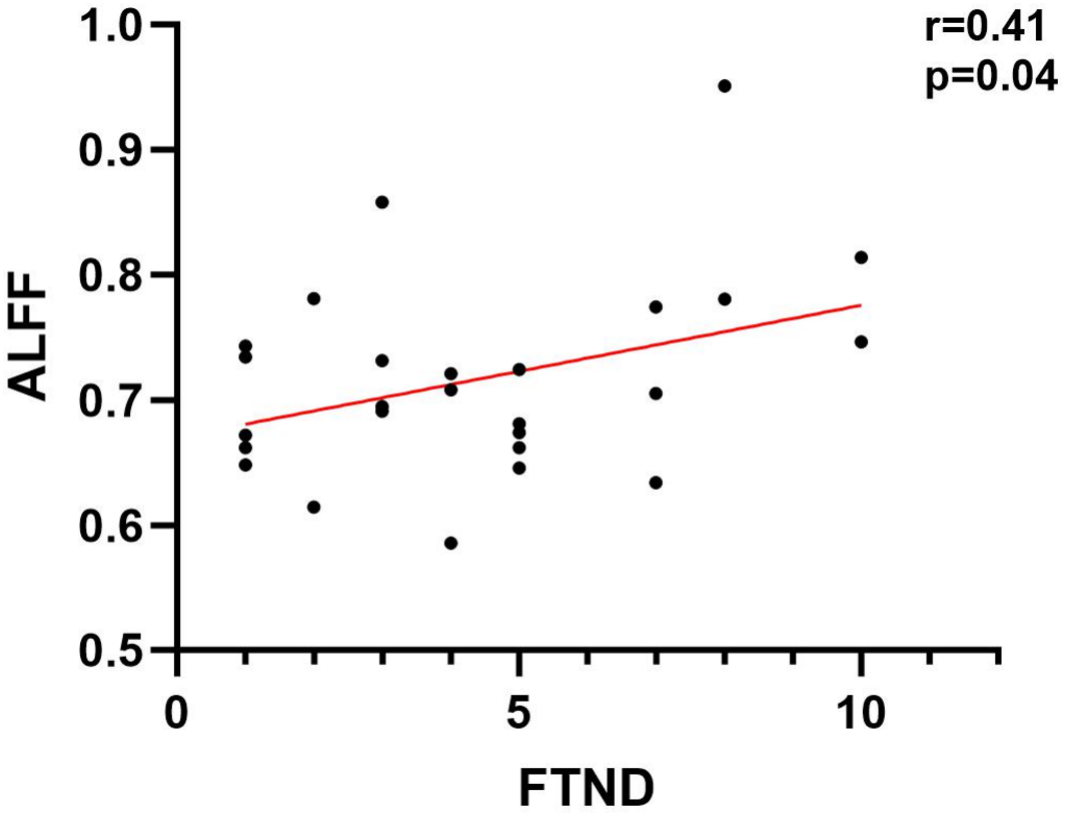
The ALFF values in the right precentral gyrus are correlated with FTND in the HA‐SM group.

The correlation analysis revealed that the ALFF values in the right precentral gyrus were significantly correlated with FTND score in HA‐SM group (*r* = 0.41, *p* = 0.04). No significant correlation was found between ALFF with other clinical data (age onset of smoking, smoke years and pack‐year) (Figure 3).

## 4 Discussion

Our study aimed to investigate the effects of smoking and HA exposure on brain activity using ALFF and to explore whether there is an interaction between the two factors. We found that, compared to nonsmokers, smokers showed enhanced ALFF values in various brain regions, including the right superior frontal gyrus, right middle frontal gyrus, right inferior frontal gyrus (triangular and opercular parts), right middle cingulate cortex and right precentral gyrus. Compared to the SL group, the HA group exhibited increased ALFF values in different brain regions, including the right putamen, right insula, right inferior frontal gyrus (triangular and opercular parts), right middle temporal gyrus, right precentral gyrus, right inferior temporal gyrus and right fusiform gyrus. Additionally, we also found an interaction between smoking and high altitude in the right precentral gyrus.

### 4.1 Brain Activity Changes Associated With the Main Effect of Smoking

Compared to nonsmokers, smokers exhibited increased ALFF in the right frontal lobe, right middle cingulate cortex and right precentral gyrus. The prefrontal cortex (PFC) is a critical brain region for executive functions. It serves as the primary node of the central executive network, responsible for task selection and behavioural control [20]. Executive functions orchestrate high‐order mental activities, enabling goal setting and the execution of goal‐directed actions [21]. In addition, PFC plays vital roles in the assessment of rewards and the formation of reward‐associated memories, which can hold on reward information processing on line, so that it can be rapidly integrated with and updated other information, in order to guide behaviour [22]. In order to respond to motivational salience and reward expectation, supervisory functions of the PFC may be down‐regulated in smoking addiction [23]. Franklin et al. [24] demonstrated through perfusion fMRI studies that smoking selectively damages the frontal lobes. Moreover, a meta‐analysis revealed that chronic smokers experience a reduction in bilateral prefrontal cortex volume [8]. Some studies have found that smoking can lead to changes in ALFF in the PFC and that these changes are associated with smoking severity, which is consistent with our findings [13, 25]. The middle cingulate cortex (MCC), known for its rich dopaminergic innervation and involvement in reward functions, is activated during noxious stimulation [26]. The MCC is a key component of the cingulate‐insular pathway, which modulates and sustains nociceptive hypersensitivity in the absence of conditioned noxious stimuli [27]. Consistent with our previous research, smoking can activate the precentral gyrus [28]. Neuroimaging studies have shown that the premotor cortex, essential for biological motion perception, is activated during motion observation [29], and it integrates auditory and visual action information [30]. Wetherill et al. [31] demonstrated using machine learning models that the sensorimotor network plays a role in the development and relapse of addictive behaviours. Due to the activation of these distinct brain regions by smoking, smokers exhibit enhanced decision‐making and execution abilities related to smoking. Despite being aware of its harmful effects, smokers find it challenging to resist the impulse to smoke.

### 4.2 Brain Activity Changes Associated With the Main Effect of High Altitude

Compared to SL populations, individuals living at HA exhibit increased ALFF values in the putamen, insula, inferior frontal gyrus, middle temporal gyrus, inferior temporal gyrus, rolandic operculum and fusiform gyrus. Hypoxia strongly stimulates the synthesis of vascular endothelial growth factor, and studies have shown that prolonged exposure to hypoxic environments leads to a significant increase in cerebral capillary density in rodents. This adaptation allows the brain to maintain sufficient oxygen and nutrient supply, mitigating damage caused by hypoxia [32]. Glial cells, which are sensitive to hypoxia [33], play critical roles in maintaining the homeostasis of the nervous system, supporting and nourishing neurons and regulating neural signal transduction [34]. Research by Vigneau‐Roy et al. [35] demonstrated a positive correlation between ALFF values of intrinsic brain activity and vascular density. Therefore, the increased ALFF observed in HA populations may be related to the enhanced vascular density in the brain. Additionally, in HA environments, high‐altitude adaptation in the cardiovascular and respiratory systems influences their corresponding control centres in the brain via afferent feedback [5]. Hypobaric pressure and cold temperatures are two additional significant factors that may contribute to changes in intrinsic brain activity among HA populations [36]. Consistent with our findings, Zhang et al. [37] discovered that, compared to short‐term HA migrants, native HA residents exhibited increased ALFF values in the putamen and fusiform gyrus. These regions may play critical roles in adapting to hypoxia. Moreover, ALFF values in the putamen and fusiform gyrus were negatively correlated with haemoglobin levels and haematocrit, indicating that ALFF changes can reflect physiological adaptations to high‐altitude environments [36]. Additionally, Zhang et al. [38] also found decreased voxel‐mirrored homotopic connectivity (VMHC) values in the precentral gyrus, along with reduced functional connectivity between the precentral gyrus and frontal gyrus. This reduction may be associated with genetic mutations that influence the regulation of specific brain regions [38]. Zhang et al. [14] found that compared to the low altitude group, the HA group exhibited significantly reduced GM volume in the insula and temporal lobe, as well as increased ALFF values in the temporal lobe, which aligns with our findings. Reduced GM volume is a quantitative indicator of brain atrophy, reflecting neuronal loss and cortical thinning. The temporal lobe is a key brain region involved in working memory, language, emotions and other cognitive domains [39]. Temporal lobe damage is closely linked to cognitive dysfunction, which may be related to metabolic changes in brain tissue following hypoxia [40]. Animal experiments by Ji et al. [41, 42] revealed that chronic hypoxia exposure in rats led to increased lipid peroxides, free radicals, and oxidized glutathione. Whether similar changes occur in human brain tissue remains to be investigated. The insula plays a critical role in respiratory control and perception [43], regulating respiratory movements to help reduce hyperventilation in HA residents [44]. Changes in the insula may also affect heart rate and blood pressure [45], aiding HA residents in better adapting to hypoxic environments.

### 4.3 Interaction Between Smoking and High Altitude

Our research shows that among nonsmokers, HA individuals exhibited increased precentral gyrus activity compared to SL individuals. Furthermore, in HA individuals, smokers demonstrated higher ALFF values in the precentral gyrus compared to nonsmokers, indicating a synergistic effect of smoking and high‐altitude exposure on the precentral gyrus. The precentral gyrus is part of the premotor cortex [46], which is also referred to as the action observation network. It plays a critical role in the visual processing of actions and is capable of handling complex arm and hand movements [47, 48]. Previous studies have shown that the premotor cortex is highly active during action observation and integrates visual and motor information. It links instructional cues with specific movements and selectively processes biological motion information, such as observing smoking behaviours in other smokers [49]. This may contribute to the habitualization of smoking behaviours in smokers. On the other hand, the precentral gyrus is crucial for controlling respiratory muscles [50]. Research has shown that the cerebrovascular reactivity of the precentral gyrus is reduced in HA residents [51], aligning with the hypometabolism hypothesis [52]. Hypometabolism is thought to develop as a mechanism to cope with hypoxic stress during HA adaptation, conserving energy and maximizing the use of limited oxygen. The increased ALFF observed in HA residents may enhance respiratory control, facilitating better adaptation to the hypoxic environment. In addition to this, we also find that the ALFF values in the right precentral gyrus are correlated with FTND in the HA‐SM group. Although there is a correlation between the two, an important factor to consider is the interindividual differences in nicotine metabolism. Changes in nicotine metabolism may lead to different levels of nicotine exposure, even between individuals with similar smoking behaviours. This change in nicotine metabolism, in turn, may have a different effect on an individual's brain function, potentially affecting ALFF values in brain regions involved in motor control and addiction.

Nicotine exposure and chronic hypoxia at high altitudes may interact to alter brain activity through their effects on neurotransmitter systems and neuroplasticity. Nicotine primarily modulates the dopaminergic system by stimulating nicotinic acetylcholine receptors (nAChRs), leading to increased dopamine release in regions associated with reward and motor control, such as the striatum and precentral gyrus [53]. Chronic nicotine use has also been shown to alter glutamatergic transmission, enhancing synaptic plasticity but potentially leading to excitotoxic effects over time [54]. In parallel, hypoxia can independently impact neurotransmitter balance, leading to dopaminergic dysregulation and changes in synaptic plasticity mechanisms, such as long‐term potentiation and dendritic remodelling [55]. These combined effects may explain the altered ALFF values observed in the right precentral gyrus, a region involved in motor control and habit formation, suggesting that prolonged exposure to both smoking and high altitude could induce neuroadaptive changes in motor‐related circuits. Additionally, hypoxia‐induced oxidative stress and neuroinflammation may further exacerbate neurofunctional alterations, potentially contributing to the observed changes in spontaneous brain activity [56]. Future studies incorporating molecular and neurochemical imaging techniques could provide deeper insights into the precise mechanisms underlying these interactions. Furthermore, the interaction between smoking and high‐altitude exposure revealed in this study provides a strong rationale for developing altitude‐specific smoking cessation programmes and policies. Based on the study's findings, precentral gyrus could be a potential transcranial stimulation target to treat HA smoking addiction. Focused ultrasound that is reported to both activate and inhibit neural activity could be a promising intervention approach [57]. In addition, medications that enhance oxygen utilization or reduce hypoxia‐related stress could be tested in combination with traditional cessation therapies.

### 4.4 Limitations

Although our study provides new insights into the neurobiological interactions between smoking and high‐altitude exposure, it has several limitations. First, all our participants were males, and the smoking rate among males is much higher than that among females. However, it has been reported that gender influences the brain function among smokers [58]. Therefore, in future, studies with participation of female subjects may provide more comprehensive results. Second, our high‐altitude participants were all from one single region (2260 m), and we did not investigate the effects of different altitudes. Future studies could further explore the differences in brain function associated with varying altitudes. Third, alcohol consumption may affect brain activity and could be a potential confounding factor. Future research should consider alcohol consumption to better control for its potential impact. Lastly, we did not assess participants' cognitive functions. Previous research has reported that chronic hypoxia induced by high‐altitude environments can lead to extensive cognitive impairments [14]. In future studies, we plan to collect participants' cognitive scores and evaluate their correlation with brain activity.

## 5 Conclusion

In summary, our study is the first to explore the combined effects of HA exposure and smoking on intrinsic brain activity using resting‐state functional MRI. Our findings indicate that both smoking and HA exposure can influence spontaneous brain activity, with a notable interaction between the two factors in altering brain function. This research may provide a neuroimaging‐based explanation for substance addiction in high‐altitude populations and offer new insights into understanding high‐altitude adaptation.

## Ethics Statement

This study was approved by the Ethics Committee of Qinghai Provincial People's Hospital, and written informed consent was obtained from all participants.

## Conflicts of Interest

The authors declare no conflicts of interest.

## Data Availability

The datasets presented in this article are not readily available because of the privacy of all the participants. Requests to access the datasets should be directed to the corresponding authors.
